# Alkaline Materials and Regenerative Endodontics: A Review

**DOI:** 10.3390/ma10121389

**Published:** 2017-12-05

**Authors:** Bill Kahler, Nadia Chugal, Louis M. Lin

**Affiliations:** 1 The School of Dentistry, The University of Queensland, Brisbane 4072, Australia; 2 School of Dentistry, UCLA, CHS A3-075, Los Angeles, CA 90095-1668, USA; nchugal@dentistry.ucla.edu; 3 College of Dentistry, New York University, 345 East 24th Street, New York, NY 10010, USA; lml7@nyu.edu

**Keywords:** regenerative endodontics, immature root, alkaline materials, disinfection, calcium silicates

## Abstract

Periapical health is the primary goal of endodontic treatment in mature and immature teeth. In addition, the goals of treatment of immature teeth with arrested root development include root growth to length and maturation of the apex, as well as thickening of the canal wall. These goals are valid for immature teeth that have been subjected to trauma and dental caries or that are the result of developmental anomalies that expose the tooth to the risk of pulp necrosis and consequently result in the cessation of root maturation. Regenerative endodontic procedures (REPs) have been described as a “paradigm shift” in the treatment of immature teeth with pulp necrosis and underdeveloped roots, as there is the potential for further root maturation and return of vitality. Treatment with REPs is advocated as the treatment of choice for immature teeth with pulp necrosis. REP protocols involve the use of alkaline biomaterials, primarily sodium hypochlorite, calcium hydroxide, mineral trioxide aggregates and Biodentine, and are the essential components of a successful treatment regimen.

## 1. Introduction

The development of pulp necrosis subsequent to trauma or extensive caries places the immature tooth at risk of cessation of root growth in length, the thickening of root canal walls and the maturation of the apex. Consequently, the tooth becomes structurally weakened in terms of withstanding masticatory forces and becomes susceptible to cervical root fracture [1]. 

Traditional methods for the treatment of these teeth include calcium hydroxide apexification, which requires multiple visits over an extended period of time. This results in a delay of root canal obturation and placement of permanent restoration [2,3]. Although the evidence is equivocal, this prolonged exposure to calcium hydroxide may predispose the tooth to increased susceptibility of cervical root fracture [4].

Recent advancements in dental materials, such as the development of calcium silicate cements, have made it possible to implement a barrier technique with mineral trioxide aggregates (MTAs) in a one-step apexification procedure. This approach has resulted in a greatly shortened treatment time and favourable treatment outcome [5]. However, neither multi-appointment apexification with calcium hydroxide or one-step apexification with the MTA barrier technique allow for further root growth in length, maturation of the apex or root wall thickening. As a result, the potential to strengthen the developing tooth root is abrogated. With the introduction of regenerative endodontic procedures (REPs) in 2001, a biologically based framework was presented in which further root maturation can occur [6].

## 2. Regenerative Endodontics

Regenerative endodontics is an exciting and rapidly developing field in the treatment of immature teeth with infected root canals and arrested root development. REPs have been described as a “paradigm shift” in the management of these teeth and can result in continued root lengthening, wall thickening and apical closure [7,8,9]. 

Regenerative endodontics has been defined as “biologically based procedures designed to replace damaged structures, including dentin and root structures, as well as cells of the pulp-dentin complex” [10]. Dental stem cells capable of differentiating into odontoblast-like cells and regenerative endodontic therapy can promote thickening of the canal walls and continued root development of immature permanent teeth with necrotic pulps. The biological concept of regenerative endodontics involves the triad of stem cells, scaffold platforms and signaling molecules [11]. The clinical considerations for regenerative endodontic protocols are (1) disinfection of the root canal system; (2) provision of a scaffold in the form of a blood clot that forms after laceration of the periapical tissue to induce bleeding and introduce mesenchymal stem cells within the root canal; and (3) an adequate coronal seal to prevent reinfection [12,13,14].

**Figure 1:**
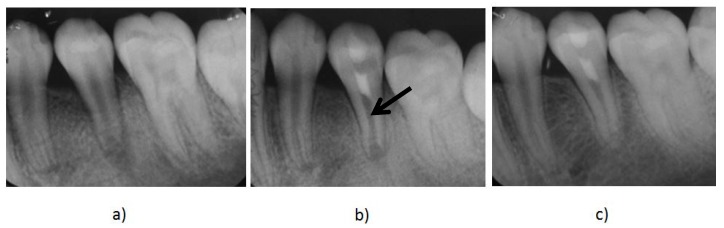
(**a**) A preoperative radiograph of an immature mandibular second premolar with pulp necrosis and apical periodontitis. (**b**) Immediate post-treatment radiograph of the tooth after the treatment with regenerative endodontic procedures (REPs). The mineral trioxide aggregate (MTA) is the radiopaque intracanal barrier (black arrow) that was placed over the induced blood clot that acted as a scaffold to the introduced stem cells promoting further root development. (**c**) A follow up radiograph taken 12 months after treatment shows further root maturation and evidence of thickening of the canal walls and apical closure.

The current recommended clinical protocol for REPs is described by the American Association of Endodontists (AAE) [14]. Figure 1 shows the management with REPs of a mandibular premolar tooth diagnosed with a necrotic pulp and apical periodontitis using the AAE guidelines [14].

The importance of root canal disinfection in REPs is well known [15,16,17]. Disinfection of the root canal system is thought to be critical for the success of REPs, as infection prevents regeneration and/or repair, as well as stem cell activity [15,17]. For most teeth treated with REPs, this involves disinfection with sodium hypochlorite at the first appointment followed by 1–4 weeks of application of intracanal medicament calcium hydroxide. At the second and/or final appointment, the blood clot is invoked following the introduction of stem cells of the apical papilla (SCAP) into the canal. A layer of MTA or Biodentine is then used to create a barrier over the blood clot.

The alkaline irrigants (sodium hypochlorite), medicaments (calcium hydroxide) and bioceramic materials (MTA or Biodentine) are integral parts of current REP clinical protocols. These essential materials used in REPs each exert different biological properties that can influence outcomes. Therefore, the purpose of this paper is to review the properties and applications of alkaline materials as applied in REPs. 

## 3. Alkaline Materials Used in REPs

A recent systematic review of the clinical protocols applied in REPs identified several alkaline materials used [18]. Sodium hypochlorite was employed as an irrigant in 97% of clinical studies. Calcium hydroxide was used in 28% of cases as intracanal medicament as an intracanal barrier, and mineral trioxide aggregate was used in 85% of clinical studies employing REPs [18]. The most recent study also identified other bioactive endodontic cements used [19]. Three studies used Biodentine [20,21,22], one used calcium-enriched material (CEM; BioniqueDent, Tehran, Iran) [23], and another used either MTA (Dentsply, York, PA, USA) or EndoSequence Bioceramic putty (Brasseler, Savannah, GA, USA) [24]. 

### 3.1. Sodium Hypochlorite (NaOCl)

Sodium hypochlorite (NaOCl) has been used as a disinfecting agent in the majority of the reported cases. NaOCl is an alkaline material with a pH ranging from 10.9 to 12 [25]. The guidelines suggest disinfection with NaOCl using a technique that minimizes the possibility of its extrusion into the periapical space. Some of the suggested techniques to this effect include the use of an irrigating needle with a closed end and side-vents, or EndoVac [14]. A volume of 20 mL/canal of 1.5% NaOCl is advised for a total of 5 min of exposure to NaOCl. Subsequently, irrigation is completed with saline or C_10_H_16_N_2_O_8_ (EDTA) (20 mL/canal for 5 min) with the irrigating needle positioned about 1 mm coronally from the root end to minimize cytotoxicity to the stem cells in the apical tissues [14]. It has been shown that EDTA irrigant stimulates the release of growth factors contained in the dentine matrix [26,27,28].

A recent review reported that 97% of clinical studies used sodium hypochlorite as either the only irrigant or in combination with other irrigants [18]. Another study reported that 63% of all REP cases were irrigated with 3% NaOCl, 36% of cases were irrigated with 5–6% NaOCl, and only 1% of cases were disinfected with 1% NaOCl, as there was no standardized protocol [29]. 

Lower concentrations of NaOCl are now recommended, as a 6% concentration has been shown to significantly decrease the survival of SCAP [30,31]. It was demonstrated that a concentration of 1.5% NaOCl has minimal destructive effects on SCAP [31]. Furthermore, the use of 17% EDTA resulted in increased survival expression of SCAP, as well as partial reversal of the deleterious effects of NaOCl [31].

### 3.2. Calcium Hydroxide

The earliest case reports describing the disinfection protocol in the treatment with REPs involved medicating the canals with a combination of antibiotics [6,32]. However, other earlier reports advocated the use of calcium hydroxide exclusively [7,33]. The vast majority of published cases seem to have employed a combination of antibiotics as the preferred intracanal medicament [18]. However, these antibiotic pastes have been shown to be cytotoxic to the survival of SCAP in concentrations equal to or higher than 1 mg/mL in in vitro studies [34,35]. In contrast, the disinfection of root canal space with calcium hydroxide promoted the proliferation of SCAP [34,35]. Calcium hydroxide also increased the release of growth factors from dentine, whereas antibiotic pastes negatively influenced growth factor release after the use of EDTA [27,28]. The AAE clinical considerations for a regenerative endodontic procedure advocate the use of either a combination of antibiotic paste or calcium hydroxide paste [14]. 

A retrospective radiographic study employing a quantitative analysis of teeth treated with REPs showed that teeth medicated with calcium hydroxide had a significantly greater increase in root length than the teeth medicated with the combination antibiotic paste [36]. Importantly, teeth treated with the combination antibiotic paste had significantly greater increases in root canal wall thickness [36]. The placement of calcium hydroxide within the canal appeared to favourably affect the outcome of the treatment. When calcium hydroxide was restricted to the coronal half of the root canal, as demonstrated radiographically, the median increase in the dentinal wall thickness was 53.8%. This contrasted with just a 3.3% increase when calcium hydroxide was present in the apical half of the root canal. The percentage change in root length was not affected in either of these different clinical protocols [36]. The results of a cohort study of 12 teeth medicated with a triple antibiotic paste showed apical closure in 66.7% of cases, increased root wall thickness in 41.7%, and increased root length in 41.7% of cases. In comparison, 11 teeth medicated with calcium hydroxide showed apical closure in 54.5% of cases, increased root canal thickness in 45.4%, and increased root length in just 27.3% of cases [37]. These findings were considered comparable outcomes, and hence, the application of either medicament is supported for use in the REP [14]. 

### 3.3. Mineral Trioxide Aggregate (MTA)

Major components of MTA powder are tricalcium silicate, dicalcium silicate, bismuth oxide, calcium sulfate dehydrate or gypsum and tricalcium aluminate. For clinical applications, MTA is mixed with saline or distilled water [38]. This aqueous mixture results in the release of calcium hydroxide and subsequent calcium silicate hydrate formation, which eventuates into a poorly crystalized and porous solid gel [39]. The precipitated calcium produces calcium hydroxide that confers alkaline properties to MTA after hydration [40]. MTA is a bioactive material, as calcium hydroxide is, and associated calcium ions allow for cell attachment and proliferation [41,42]. The high pH confers antibacterial properties [43,44]. Hard tissue-producing cells differentiate and migrate onto the MTA surface, forming a biological seal [45,46,47].

In REPs, MTA paste is placed over the blood clot or a scaffold and acts as a coronal barrier to prevent coronal leakage and ingress of microorganisms. It was reported that 85% of REP studies used MTAs for this purpose [18]. MTA has a pH of 10.2 after mixing with water, which rises to 12.5 after 3 h [48]. Furthermore, MTA has been shown to be a good sealing material [49,50] and is less cytotoxic than other materials used in dentistry [51]. In bacterial leakage investigations, no significant difference has been shown between MTA and glass ionomer cement (GIC) [52,53]. However, MTA has better marginal adaption compared to GIC [54]. Numerous studies have shown that MTA is a biocompatible material [55,56] and is also bio-inductive [57]. All these properties make MTA the material of choice for use as an intracanal barrier matrix over the blood clot. A disadvantage of MTA is discoloration of the coronal dentine when placed in the canal [58]. Many studies have shown that discoloration is a significant negative outcome following REPs [59]. This is of particular concern for traumatized anterior teeth, as appearance and pleasing aesthetics are patient-centered outcomes. 

### 3.4. Biodentine

Biodentine is a bioceramic cement in the same family of compounds as MTA. The material is composed of tricalcium silicate, dicalcium silicate, zirconium oxide, calcium carbonate, calcium oxide and iron oxide. It is mixed with a hydrosoluble polymer and calcium chloride to decrease the setting time [38]. Biodentine is an alkaline cement with a pH range of 11.7–12.3 [60]. This material has also been shown not to be cytotoxic to pulp fibroblasts [61]. The dentine bridge formation formed by MTA and Biodentine was found to be comparable [62]. Biodentine can also be used as an intracanal barrier over the blood clot [20,63] and has been shown to stain teeth less than MTA [64,65,66]. Further studies on Biodentine effectiveness in REPs are needed prior to its recommendation for widespread use. It has been used in three studies that employed REPs [19].

### 3.5. Calcium-Enriched Material (CEM)

CEM consists primarily of calcium oxide, sulfur trioxide, phosphorous pentoxide and silicon dioxide, which is mixed with water [67]. It is an alkaline material with a pH of 10.7 [68]. CEM has a shorter setting time than MTA and similar sealing ability and biological properties [67]. CEM has only been used in one study employing REPs [23].

### 3.6. EndoSequence Bioceramic Putty

EndoSequence Bioceramic putty is a ready-to-use putty consisting primarily of calcium silicate, monobasic calcium phosphate, zirconium oxide, and tantalum oxide. The manufacturer states that the moisture present in the dentinal tubules is sufficient for the material to set [67]. EndoSequence Bioceramic putty has a pH greater than 12; thus it is an alkaline material [69]. This material has a similar sealing ability and biological properties to MTA [67]. EndoSequence Bioceramic putty has only been employed in one study employing REPs [24]. 

## 4. Future Prospects

Regenerative endodontics has gained increased attention in the provision of biologically based treatment in teeth with arrested root development [11]. The success of the procedure has opened up avenues for the treatment of adult permanent teeth to be managed with REPs instead of conventional endodontic therapy [70,71,72,73]. With advances in stem cell-based pulp tissue engineering, it is possible that REPs will be increasingly incorporated into clinical practice [11]. 

## 5. Conclusions

Alkaline materials play an integral role in REPs as disinfecting irrigants, intracanal medicaments and cements used for the creation of an intracanal barrier over the blood clot. Lower concentrations of sodium hypochlorite are recommended over those used for conventional endodontic protocols, to minimize its deleterious effects on Stem Cells of the Apical Papilla (SCAP) cells. Calcium hydroxide encourages further root development when placed in the coronal half of the root canal. When used as an intracanal barrier, MTA is associated with a notable incidence of crown discolouration. Biodentine appears to have less staining effects on dentine than MTA. However, further studies are needed to demonstrate its efficacy and long-term effects. As our knowledge in pulp biology and dental biomaterials advances, the treatment of immature permanent teeth with necrotic pulp will be improved accordingly.
